# Massage increases satellite cell number independent of the age‐associated alterations in sarcolemma permeability

**DOI:** 10.14814/phy2.14200

**Published:** 2019-09-08

**Authors:** Emily R. Hunt, Amy L. Confides, Sarah M. Abshire, Esther E. Dupont‐Versteegden, Timothy A. Butterfield

**Affiliations:** ^1^ Department of Rehabilitation Sciences, Center for Muscle Biology University of Kentucky Lexington Kentucky

**Keywords:** IgG, Pax‐7, permeability, sarcolemma, satellite cells

## Abstract

Massage is a widely accepted manual therapy used to modulate the inflammatory response of muscle and restore function, but prolonged compression of muscle potentially causes overt injury and damage to muscle fibers. Therefore, a balance exists between the positive effects of massage and the induction of mechanical damage and injury. In addition, skeletal muscle of aged individuals displays increased stiffness, and therefore, the response to massage is likely different compared with young. We hypothesized that the aged skeletal muscle exhibits increased sarcolemmal permeability when subjected to massage compared with young skeletal muscle. Male Brown Norway/F344 rats, 10 and 30 months of age, were each divided into control, non‐massaged (*n* = 8) and massaged (*n* = 8) groups. The right gastrocnemius muscle received one bout of cyclic compressive loading for 30 min at 4.5 N as a massage‐mimetic. Muscles were dissected and frozen 24 h after massage. *A*lterations in sarcolemma permeability were quantified by measuring the level of intracellular IgG within the muscle fibers. Immunohistochemistry was performed to determine IgG inside fibers and Pax7+ cell number as an indicator of stem cell abundance. Average IgG intensity was not different between control and massaged animals at either age. However, a significant shift to the right of the density histogram indicated that massaged animals had more fibers with higher IgG intensity than control at 10 months. In addition, Pax7+ cell number was significantly elevated in massaged muscles compared with control at both ages. One bout of massage did not induce overt muscle injury, but facilitated membrane permeability, which was associated with an increase in satellite cell number. Data suggest that the load applied here, which was previously shown to induce immunomodulatory changes, does not induce overt muscle injury in young and old muscles but may result in muscle remodeling. Funded by NIH grant AG042699 and AT009268.

## Introduction

Massage is a manual therapy widely used to treat a variety of musculoskeletal conditions, and is defined as the *manipulation of body tissues with rhythmic pressure and stroking for the promotion of health and well‐being*. (Galloway and Watt 2004) Clinicians use massage most frequently postexercise to enhance recovery and aid in muscle function (Tiidus 1997; Best et al. 2008; Crawford et al. 2014). Although there are numerous reports focused on human massage, the majority of evidence in support of massage efficacy remains anecdotal and there is a need for mechanistic research (Furlan et al. 2002; Shin and Sung 2015). To determine the mechanisms underlying the physiological effects of massage on muscle tissue, there has been an increased reliance on the use of animal models where tuneable loads and appropriate controls can be more easily used.

Previous studies using a rabbit model provide evidence that massage can increase peak torque and improve function of muscle when applied postexercise (Butterfield et al. 2008). Further investigations have revealed an optimal dose for massage that includes a set frequency and duration for application of the optimal load, as well as the timing for massage after damaging exercise (Haas et al. 2012, 2013a, 2013b). As such, massage (applied as cyclic compressive loading, CCL, in the laboratory setting) modulates the expected postexercise inflammatory response and limits the detrimental effects of damaging exercise by attenuating the pro‐inflammatory cascade (Waters‐Banker et al.  2014a). In addition, massage alters the inflammatory environment of unperturbed muscle in a load‐dependent manner in the massaged limb and in a load‐independent manner in the contralateral limb (Waters‐Banker et al. 2014b). These studies indicate that massage is an immunomodulatory intervention inducing cellular responses around an optimal load.

When applied immediately following constrained and damaging eccentric exercise in rabbits, massage significantly reduced the numbers of macrophages, and neutrophils within the muscle (Butterfield et al. 2008; Haas et al., 2013a). This reduced endomyseal inflammation was associated with accelerated recovery and return of normal muscle form, contractile function (Butterfield et al. 2008; Haas et al.  2013a, 2013b) and passive muscle properties (Haas et al. 2012). Although massage may have limited the secondary injury that occurs within 24 h of the exercise bout (Haas et al., 2013a), the mechanisms whereby cells respond to loading using CCL as a massage‐mimetic, have not been determined.

Prolonged compression of muscle will eventually lead to a load‐dependent cell injury induced through a cellular mechanism during ischemia (Wang et al. 2005; Stekelenburg et al. 2007). Cellular injury is an immune mediated event, whereby the pro‐inflammatory response following the tissue perturbation induces delayed secondary damage to the cell membrane (Tidball 2005). Mechanically induced cell injury can occur when the external loads cannot be attenuated internally, and exceed the internal load bearing capacity of the cell. If the magnitude of cell membrane deformation exceeds a given strain magnitude, a hole or tear can form in the cell membrane leading to a simultaneous influx of macromolecules and ions with an efflux of cytoplasmic contents (Tidball 2005) creating an altered homeostasis that will eventually lead to the necrosis and death of the muscle cell if the membrane is not resealed (Tidball 1995). Although a mechanically induced membrane defect can be quickly sealed, the mechanical poration of the membrane can have significant effects on the neighboring cells due to the release of cellular contents into the extracellular space (Barbee 2006). The subsequent response of the tissue following membrane poration is dependent upon the severity of the pore, the extracellular environment and the type of cells in the vicinity (Barbee 2006). Satellite cells, the resident stem cells of muscle, reside between the basal lamina and cell membrane of muscle cells or fibers. Satellite cells remain mostly quiescent in adult muscle, but become activated during insult to the muscle that is most often categorized as muscle injury. Once activated, satellite cells divide and aid in the recovery of the injured muscle cells to repair damaged muscle (McCarthy et al. 2011; Fry et al. 2014).

To date, the beneficial effects of massage have been investigated in skeletal muscle of young animals and humans, but the response of aged muscle to a mechanical stimulus such as massage applied by CCL remains unknown. Aged skeletal muscle has elevated fibrosis and a stiffer extracellular matrix compared to young muscle (Brack et al. 2007). These age‐related changes in skeletal muscle structure leave the fibers more susceptible and less responsive to injury (Brooks and Faulkner 1994; Grounds 1998; Stearns‐Reider et al. 2017). To what extent satellite cells play a role in the response to massage and/or membrane mechanoporation is currently unknown. Therefore, the purpose of this study was to determine if a dose of massage which is immunomodulatory in healthy, young unperturbed muscle, will induce sarcolemma injury and satellite cell number accretion in young and aged skeletal muscle.

## Methods

### Animals

Thirty‐two male Brown Norway/F344 rats were obtained from the Aged Rodent Colony of the National Institutes on Aging (Bethesda, MD) and housed in the Division of Laboratory Animal Services at the University of Kentucky. Rats were housed in pairs on a 12:12 light/dark cycle with access to water and food ad libitum. Rats were stratified into two groups by age: young rats, 10 months of age (*n* = 16); and aged rats, 30 months of age (*n* = 16). Rats were then randomly divided into two groups: non‐massaged, control (C)) and massage (M) for both young adult, 10 months old (10C, *n* = 8 and 10M, *n* = 8) and aged, 30 months old (30C, *n* = 8 and 30M, *n* = 8). All procedures were approved by the Institutional Animal Care and Use Committee at the University of Kentucky,

### Application of the massage‐mimetic

Rats in the 10M and 30M groups were anesthetized and received one bout of a CCL, whereas 10C and 30C rats were anesthetized only. The massage‐mimetic was applied as previously described (Waters‐Banker et al. 2014b; Miller et al. 2018). Briefly, rats where placed on a mesh sling in a left lateral recumbent position with the right hindlimb secured to a small platform by self‐adherent Coban™ tape encircling the talocrural joint/midfoot. This placed the lateral aspect of the right gastrocnemius muscle facing superiorly for the application of a massage‐mimetic by a custom fabricated device described previously (Waters‐Banker et al. 2014b). The cyclic compressive loading device is a spring‐loaded mechanism containing a cylindrical roller that will glide over the muscle mass. The roller will be displaced in response to the normal force being exerted back onto the roller from the muscle, and the forces are measured in real time. For massage application, the roller was placed on the skin overlying the right gastrocnemius muscle immediately posterior and proximal to the lateral malleolus and cycled proximal to distal and back along the length of the gastrocnemius muscle, applying 4.5 N with a duty cycle of 0.25 for 30 min. After completion of the massage treatment or control sham treatment to the right gastrocnemius, rats were placed back into their cages and allowed to recover. Twenty‐four hours following the massage treatment, the animals were euthanized via an injection of Euthasol^®^ (IP) and exsanguinated. Right gastrocnemius muscles were harvested, flash frozen in liquid nitrogen, and stored at −80°C for further analyses.

### Immunohistochemistry

**Figure 1:**
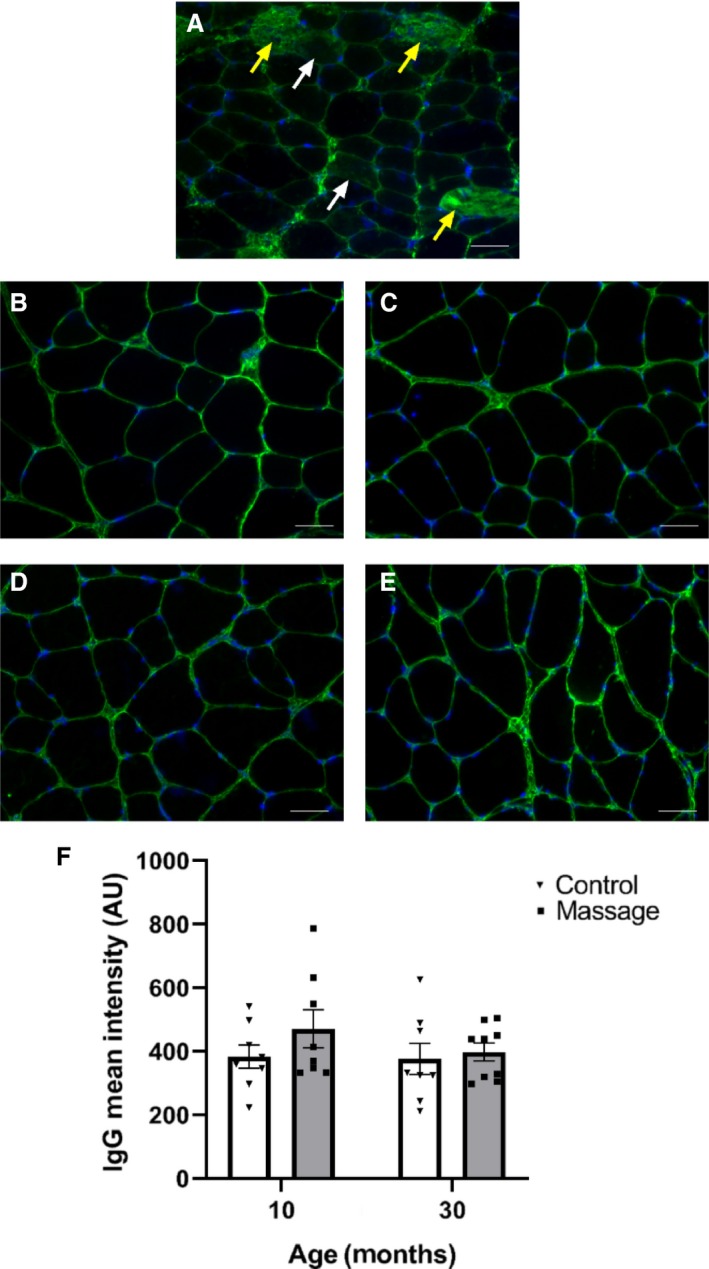
No overt damage induced by massage in young and old muscle. IgG (green) staining of tibialis anterior muscle after down‐hill running (A) demonstrating IgG infiltration into injured fibers (yellow arrows). Additional fibers exhibiting moderate to low IgG infiltration did not meet the criteria for injury (white arrows). Representative cross sections of gastrocnemius muscles immunoreacted for IgG from: (B) 10C; (C) 10M; (D) 30C; and (E) 30M. Bar graph depicting IgG mean intensity of fibers from controls (C) and massaged (M) groups of young (10 month) and old (30 month) rats (F). Bars indicate mean ± SE. There were no significant differences in IgG mean intensity.

Immunoglobulin G (*IgG): A*lterations in sarcolemma permeability were quantified by measuring the level of intracellular IgG within the muscle fibers of the massaged and control gastrocnemius muscles of young and aged rats as described previously (Huntsman et al. 2013; Boppart et al. 2013; Miller et al. 2018). IgG is the main low molecular weight protein (150 kDa) in mammalian serum, and a large (10.0 nm diameter) extracellular matrix component (Prausnitz and Noonan 1998; Tabrizi et al. 2010). Healthy sarcolemma is impermeable to large extracellular proteins, therefore the presence of IgG in the sarcoplasm of muscle fibers is indicative of increased membrane permeability (Barbee 2006). For immunohistochemistry staining, frozen gastrocnemius muscles were sectioned to 8 *μ*m thickness, fixed in ice cold acetone, and blocked in 5% bovine serum albumin in PBS. Muscle sections were then incubated with FITC‐conjugated mouse anti‐IgG (1:100) (Vector Laboratories, Burlingame CA) overnight at 4°C. Muscle sections were then cover slipped with Vectashield (Vector Laboratories). Images of muscle sections were captured with a Zeiss upright microscope (Axio Imager, M1, Zeiss, Gottingen, Germany). Five random fields were photographed using Zeiss Axiovision software v4.8, and all digital images were stored as raw images without processing. Images were visualized using fluorescence microscopy at 200× magnification to quantify IgG infiltration and count fibers. Membrane permeability was determined based on the density measurements inside the muscle fibers. The mean fluorescence intensity of each fiber was determined using Axiovision analysis software. The densitometric analysis provided a continuum of intracellular fluorescent intensity measurements for each muscle fiber, and was expressed in arbitrary units (AU). A higher fluorescence intensity indicates a greater membrane permeability and greater infiltration of IgG into the muscle fiber. For injury‐positive control tissues, IgG infiltration was measured in fibers from eccentrically‐exercised tibialis anterior muscle from a previous study (Butterfield, et al. 2005) (Fig. 1A).

**Figure 2:**
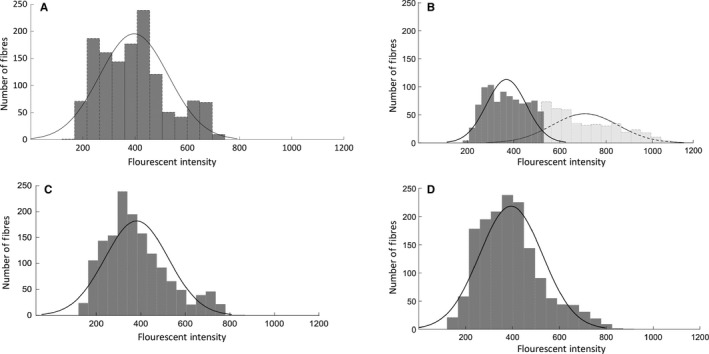
Massage has an age‐dependent effect on membrane permeability. Frequency distributions of the fluorescence intensity mean of IgG staining for gastrocnemius muscles from (A) 10C, (B) 10M, (C) 30C, and (D) 30M groups. Each frequency distribution was analyzed for a Bimodality coefficient. The young massage (10M) group was the only group to display a significant rightward shift in mean intensity distribution (BC = 0.5836).

To determine whether subsets of fibers had increased permeability, a bimodal coefficient was calculated according to Freeman et al. (Pfister et al. 2013). The fluorescent intensity data for each group were imported into MATLAB (version R2018b, Mathworks, Natick, MA) and analyzed with a series of custom written MATLAB scripts. Group data were first partitioned using a binning algorithm in MATLAB that returned the data in bins with width calculated to best reveal the underlying shape of the distribution. Mechanical deformation of tissues can result in membrane poration, and a rightward shift in the histogram output, indicating a greater subpopulation of cells exhibiting higher fluorescent intensities (Geddes et al. 2003) (Fig. 2). Therefore, to uncover the existence of any subsets of fibers exhibiting greater membrane permeability (increased fluorescent intensities), we further analyzed the distributions of the data for modality by calculating the Bimodality Coefficient (BC) for each group; a BC > 0.555 indicates a bimodal distribution (Freeman and Dale 2013).

### Pax7

Pax7 was detected by immunohistochemistry on air‐dried frozen gastrocnemius muscle sections (8 *μ*m) fixed in 4% paraformaldehyde as described previously (Miller et al. 2018). Briefly, following fixation, sections underwent an epitope retrieval protocol at 92°C using sodium citrate buffer (10 mmol/L, pH 6.5). Endogenous peroxidase activity was blocked with 3% hydrogen peroxide in PBS and sections were blocked with blocking reagent (TSA, Invitrogen, Carlsbad, CA). Sections were then incubated in Pax7 primary antibody (Developmental Studies Hybridoma Study Bank, Iowa City, IA) at a 1:100 dilution followed by incubation with a goat anti‐mouse biotin‐conjugated secondary antibody (1:1000, Jackson ImmunoResearch, West Grove, PA) and subsequently streptavidin‐ HRP (1:100) included as part of the Tyramide Signal Amplification kit (TSA, Invitrogen, Carlsbad, CA). TSA‐Alexa Fluor 594 (Invitrogen) was used to visualize antibody‐binding. Sections were counterstained with DAPI (1:10,000, Invitrogen) for nuclear detection and mounted with Vectashield fluorescent mounting medium (Vector Laboratories). Five fields per gastrocnemius muscle were visualized and imaged at 200x magnification. Pax7+/DAPI+ nuclei were counted and normalized to fiber number.

### Statistical analysis

Differences between groups were determined by a two‐way ANOVA with Bonferroni post hoc test using GraphPad Prism, version 8.0.2. All data are expressed as mean ± SE, alpha was set at 0.05 a priori.

## Results

The mean membrane permeability as measured by IgG infiltration into the muscle fibers was not altered due to massage (*P* = 0.227) or age (*P* = 0.377) and no significant interaction was observed between age and massage (*P* = 0.459) (Fig. 1), indicating that independent of the age of the animal massage did not induce damage to the muscle fibers at the load used in this study. In addition, no muscle fibers from any of our experimental groups exhibited fluorescent intensities that were close to the ones in the injured muscle (injured muscle, AU ranged 1441–3000; massaged muscles ranged 212–496, Fig. 1A–E). This indicates that 30 min of massage applied to young and old muscles using a load of 4.5 N does not induce overt fiber injury after 24 h (Fig. 1B–E).

Mean intensity values do not show whether subsets of fibers are affected differently by massage, and therefore, we determined a BC. Bimodal distribution of fiber fluorescence was observed within the 10M group (Table 1), but not for any of the other groups. To accurately differentiate between the subpopulations within the 10M group, fiber fluorescence data were fit to a Gaussian mixture model to separate the two clusters of data with unique means and standard deviations. Within the 10M group, Cluster 1 contained 67.64% of the total fibers with a mean fluorescent intensity of 368.51 ± 85.76 AU similar to the intensity measured in 10C (395.99 ± 131.75 AU), 30M (393.80 ± 134.23 AU), and 30C (381.24 ± 142.26 AU) groups. However, the second subpopulation of fibers in the 10M group showed a mean fluorescence of 708.52 ± 142.42 AU, indicating that this subset (32.65%) of fibers from the10‐month‐old rats exhibited increased membrane permeability in response to applied massage.

**Table 1:** Results of the distribution analysis showing the Bimodality Coefficient (BC) for each group

Group	Bimodality coefficient (BC)
10 C	0.4907
10 M	***0.5836*** *
30 C	0.5084
30 M	0.4636

* Indicates a significant bimodal distribution (BC > 0.5555) (Pfister et al. 2013).

**Figure 3:**
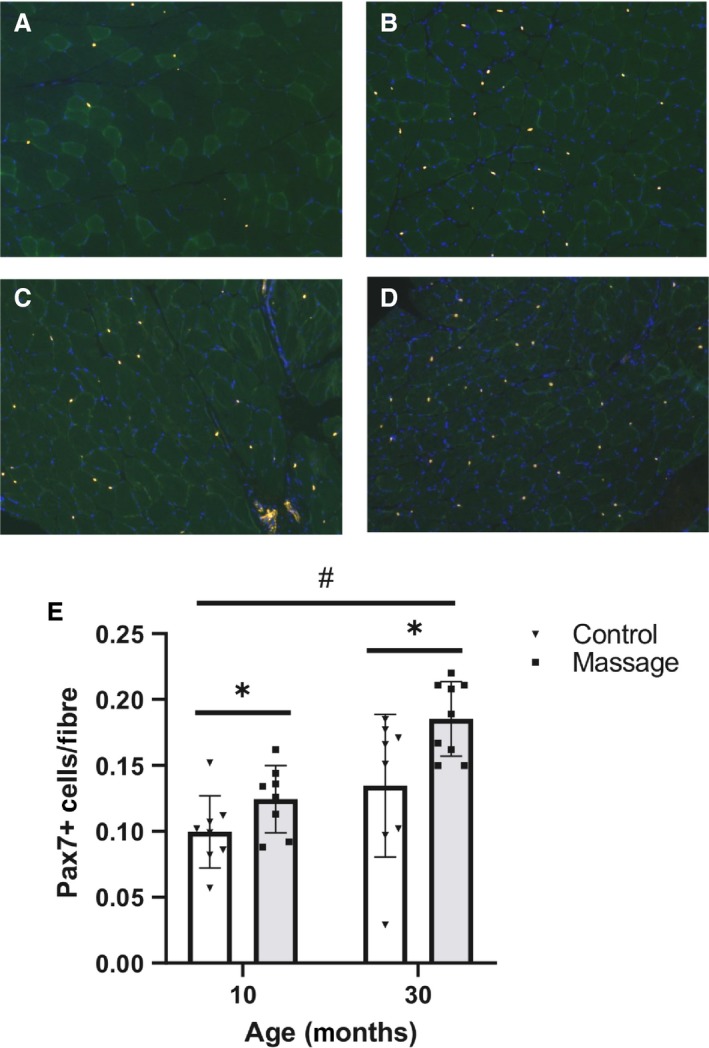
Massage is associated with higher number of Pax7 positive cells. Representative cross sections of gastrocnemius muscles positive for Pax 7 cells from: (A) 10C (*n* = 8); (B) 10M (*n* = 8); (C) 30C (*n* = 8); (D) 30M (*n* = 9). Bar graph (F) depicting average Pax7+ cells. Bars indicate mean ± SE. Pax 7+ cells were significantly increased with massage in both the 10‐month and 30‐month‐old groups compared with the respective control groups (*P* = 0.007). Pax7+ cells per fiber were significantly increased in the 30‐month‐old rats compared with the 10‐month‐old rats (*P* < 0.001). * indicates a main effect for massage; # indicates a main effect for age.

Pax 7+ cells were counted to investigate whether the higher membrane permeability with massage was associated with differences in satellite cell number (representative images are shown in Fig. 3A–D). Muscles for massaged animals had significantly more Pax 7+ cells compared with the non‐massaged control groups (main effect, *P* = 0.007), indicating that massage increased satellite cell number regardless of age (Fig. 3E). Aged animals also had significantly more Pax 7+ cells within the gastrocnemius muscle regardless of treatment (main effect, *P* < 0.001, Fig. 3E).

## Discussion

In this study, we sought to determine whether aged skeletal muscle fibers would be more susceptible to membrane poration during massage when compared to muscle fibers of young animals, when using a load optimized for young adult muscle (Waters‐Banker et al.  2014b). Muscle fibers, are continually subjected to forces internally and externally that result in immediate or delayed adaptive responses. The response of the cell to loading depends, in part, on the material and structural properties of the cell membrane, and internal cytoskeleton that provide viscoelasticity to the cell. When the force or loading rate produces a cell membrane strain that exceeds its physiological threshold, the localized membrane deformation leads to a membrane defect, or pore. This transient *mechanoporation* of cell membranes has been well characterized in many cell types (McNeil and Steinhardt 1997; Blackman et al. 2000; LaPlaca et al. 2018) often through the use of tracer dyes (Roche et al. 2009) or quantifying large macromolecules that normally remain outside of the intact cell membrane (Boppart et al. 2013). Here, we measured the intracellular signal intensity elicited from fluorescently labeled IgG as an indicator of the magnitude of transient mechanoporation, or membrane permeability following one bout of massage to skeletal muscle.

IgG is a membrane‐impermeant protein and it is therefore localized to the extracellular space in healthy skeletal muscle. However, under conditions whereby the sarcolemma is rendered “leaky” through transient mechanoporation, this large extracellular protein leaks into the muscle fiber. Using standard immunofluorescence techniques, IgG becomes an effective in vivo tracer for sarcolemmal damage (Boppart et al. 2013). Surprisingly, we observed no differences in membrane permeability between any of the groups indicating that mechanoporation between all muscles was similar independent of age and treatment. Therefore, massage as a mechanical stimulus was not injurious to muscles of young and aged rats.

Interestingly, there was a bimodal distribution of IgG abundance in young massaged animals only, not in aged. An individual muscle fiber's distance from the massage load, its morphology, and viscoelastic properties will influence cyclic strain magnitudes and subsequent cellular responses in vivo. Using an in vitro model of cell stretch, Geddes et al. (2003) demonstrated a non‐uniform response of neurons to a well‐controlled mechanical strain, and identified a subpopulation of cells with increased susceptibility to mechanoporation, a function of the mechanical properties of the cells. This is similar to our in vivo findings herein, as massage induced a mechanoporation, or increased membrane permeability in a subpopulation of muscle fibers from young adult skeletal muscle only. This is of significance for the following: the load used to massage the muscles in this study was determined to not only be optimal for enhancing muscle regrowth and remodeling following disuse atrophy in young rats of the same strain and age (Miller et al. 2018), but also as an immunomodulatory load in young rats (Waters‐Banker et al., 2014b) and an immunosuppressive load in young rabbits (Haas et al., 2013a). Furthermore, the attenuated response to resistance exercise in aged muscle compared with young (Drummond et al. 2008) may not be due to a difference in the mechanical load applied, but to other mechanisms residing inside the cell itself.

Muscle from aged rats did not respond to one bout of massage as a mechanical load with elevated mechanoporation, unlike the young muscle in this study. This could be due in part to the progressive changes in muscle morphology as the tissue ages (Frantz et al. 2010; Plate et al. 2013). Specifically, changes in the quality and quantity of the ECM in skeletal muscle would alter the viscoeleastic properties of the tissue, affecting the immediate (elastic) and time dependent (viscous) response of the muscle to applied load (Wood et al. 2014). The ECM and sarcolemma of aged skeletal muscle demonstrate different material properties, with tissue becoming stiffer as it ages (Plate et al. 2013). Along with the biomechanical properties of the ECM changing with age, older animals also exhibit higher collagen concentrations and more densely packed collagen cross‐linked fibers (Wood et al. 2014). The ECM plays a crucial role in force transfer and tissue response to mechanical load (Kjær 2004) and it is therefore feasible that aged muscle fibers are “stress‐shielded” from mechanical load. This stress shielding would result in higher strain magnitudes needed to induce muscle membrane lesions, and an attenuation of the load within the ECM, reducing the stress and strain of the fibers. We suggest that the stiffer extracellular matrix therefore is protective against mechanical loads that in young animals induce mechanoporation.

Massage was associated with a higher number of Pax7+ cells, indicating that mechanical loading, and not mechanoporation was a sufficient stimulus to increase satellite cell number in skeletal muscle, regardless of age. Due to their position outside of the muscle cell membrane, mechanosensitive satellite cells sense and respond to changes in the mechanical environment of their niche, which is composed of ECM components (Fry et al. 2017) and tethered by a cell matrix connection that includes integrins and the dystrophin‐associated glycoprotein complex (Boers et al. 2018). Therefore, satellite cells are sensitive to structural changes in the extracellular matrix and the mechanical properties of muscle and to external loads applied to muscle. Muscle stiffness increases with age and as the extracellular matrix becomes more rigid the “stemness” and differentiation of satellite cells decreases, which could have a negative impact on the muscles ability to regenerate (Gilbert et al. 2010). Specifically, an increase in aged fibroblasts found in the ECM will influence stem cell fate and the ability of satellite cells to proliferate (Stearns‐Reider et al. 2017). Satellite cells are important regulators of the extracellular matrix (Fry et al., 2015, 2017) and a depletion of the satellite cell pool can lead to increases in fibrosis (Murphy et al. 2011), while decreased regenerative response of the aged muscle is likely related to the response of satellite cells to a stiffer ECM environment (Lacraz et al. 2015). Our data show that satellite cells respond to a mechanical stimulus by increasing in number, but the function of this increase is unknown as our previous data indicate that they do not contribute to an increase in nuclear number in response to massage (Miller et al. 2018). An increase in mechanoporation in young coupled with an increase in satellite cell abundance suggests that massage is inducing mild fluctuations in the sarcolemma and extra cellular environment which are different in aged muscle. The importance of these findings needs to be further investigated.

## Conclusion

Massage is a widely used modality and understanding the physiological mechanisms that underly its effects is essential. It is important to establish how massage affects not only perturbed muscle but also healthy unperturbed skeletal muscle at all ages to determine potential implications for improving function. Here, we show that a single bout massage did not cause any significant changes in sarcolemma permeability in young and aged muscle, even though slight elevations in membrane permeability may have occurred in young in a subset of fibers. Lastly, massage is able to enhance muscle stem cell number and this may contribute to the ability of massage to aid in repair of injured muscle.

## Conflict of Interest

None declared.
